# The Role of Metacognitive Beliefs in Predicting Academic Procrastination Among Students in Iran: Cross-sectional Study

**DOI:** 10.2196/32185

**Published:** 2022-07-28

**Authors:** Yahya Safari, Nasrin Yousefpoor

**Affiliations:** 1 Department of General Paramedicine School of Paramedical Sciences Kermanshah University of Medical Sciences Kermanshah Iran

**Keywords:** procrastination, metacognitive awareness, medical students, academic training

## Abstract

**Background:**

Academic procrastination is a challenge that many students face. Metacognitive beliefs are the main cause of academic procrastination because they are one of the main reasons for students' academic failure or progress.

**Objective:**

This study aimed to determine whether and to what extent academic procrastination could be predicted based on students’ metacognitive beliefs.

**Methods:**

This descriptive cross-sectional study involved 300 students selected via stratified random sampling. Data were collected using the Procrastination Assessment Scale for Students and the Metacognition Questionnaire-30. The data analysis was done using the Pearson correlation coefficient and regression analysis to estimate the correlation coefficient and predictability of academic procrastination based on metacognitive beliefs.

**Results:**

A significant negative correlation was observed between the subscale of positive beliefs of concern and academic procrastination (*r*=–0.16; *P*<.001). In addition, the metacognitive beliefs of the participants predicted 10% of academic procrastination. The component of positive metacognitive beliefs with the β value of 0.45 negatively and significantly predicted the students’ academic procrastination (*P*<.001), whereas the component of negative metacognitive beliefs with the β value of .39 positively and significantly predicted the students’ academic procrastination (*P*<.001).

**Conclusions:**

Metacognitive beliefs can predict students' academic procrastination. Therefore, the modification of metacognitive beliefs to reduce procrastination is suggested.

## Introduction

Academic procrastination is a challenge faced by many individuals and organizations [1], and it is a major cause of learners’ failure to attain academic achievement [2]. A study conducted by Kagan et al [3] involving 265 students from different universities, departments, and classes showed that among students with individual characteristics of perfectionism and compulsive obsession, 67 (25%) showed some a degree of academic procrastination, which disrupted their ability to learn [3]. *Academic procrastination* could be defined as a student delaying their studies until the night before an exam, which affects the student's academic achievement. In academic settings, students have specific tasks to perform, such as writing term papers; studying for exams; reading assignments; and performing academic, administrative, and attendance tasks. However, for one reason or another, the completion of these tasks is often postponed. The general propensity to engage in such dilatory behavior is called *academic procrastination* [4]. The main consequences of academic procrastination are poor educational performance and the feeling of negative emotions, such as guilt and shame. Notably, procrastination is not always an issue, since not completing a task is in some cases better than doing it incompletely [5]. Academic procrastination emerges as a deliberate delay in a practical course of study and tasks such as reading, article writing, and preparing for an exam. Academic procrastination is an irrational desire to delay the completion of a homework assignment or other academic tasks. Under such circumstances, students lack the motivation for academic activities at certain times despite the intention to complete them. Consequently, they fail to complete assignments to their desired level of quality within the expected time frame, which adversely affects their mental health [6].

Dysfunctional cognitive and metacognitive beliefs are considered to be the main causes of academic procrastination [7]. In general, cognitive approaches emphasize the impact of negative attitudes and beliefs on procrastination, though they fail to explain the efficacy of such beliefs through different mechanisms. Nonetheless, the metacognitive perspective of procrastination could accurately explain these processes [8]. According to Flavell, metacognition is the cognitive knowledge or process that cooperates in the assessment, review, and control of cognition, thereby tuning cognitive performance [9]. Moreover, it could be used to link and combine new information with previously learned data that are to be stored in long-term memory [10].

Most theorists believe there are 2 distinct aspects of metacognition. The first aspect is metacognitive knowledge, which refers to the knowledge one has about their cognitive processes and strategies for learning [11]. The second aspect is metacognitive regulation, which refers to different types of executive actions, such as attention, review, planning, and the identification of performance errors in terms of their impact on cognitive activity [11,12]. Therefore, procrastination is related to metacognition from 2 perspectives. First, procrastination is considered a strategy for regulating cognition. Second, procrastination is associated with negative emotions and is considered a strategy adopted by individuals to avoid and regulate negative emotions [13].

With regard to the performance of students on academic assignments, some researchers have considered cognitive elements to be the strongest predictors of learning, while others have highlighted the role of metacognitive components in this regard [14]. Studies have shown a positive correlation between procrastination and difficulties in emotional regulation [15]. Furthermore, a positive association has been reported between improper emotional regulation strategies (eg, blaming others) and procrastination. Therefore, training on emotional regulation skills could reduce procrastination [16]. Nonetheless, some studies have identified academic procrastination in 80%-90% of students [17].

In a study on this topic, Özer et al [18] reported procrastination in the process of article writing, preparation for exams, and the completion of weekly assignments in 46%, 27%, and 30% of students, respectively. According to Özer and Sackes [19], 38% of students were severe procrastinators, although Walters [11] reported a reverse correlation between adopting certain metacognitive beliefs and procrastination. Furthermore, results obtained by Howell and Watson [20] indicated a reverse correlation between certain metacognitive beliefs and academic procrastination.

Given the previously mentioned findings and the high prevalence of procrastination among students, authorities, educational planners, and those involved in the academic system must adopt strategies for the management and reduction of procrastination in students. This could be a step toward solving the educational problems of learners at different levels of study. This study aimed to determine the role of metacognitive beliefs in the prediction of academic procrastination. The main question of the study is the following: to what extent do metacognitive beliefs predict academic procrastination?

## Methods

### Ethics Approval

This study was approved by the ethics committee of Kermanshah University of Medical Sciences (IR.KUMS.REC.1396.446), and participants provided written informed consent for participation in this study.

### Participants and Setting

This descriptive-analytical cross-sectional study focused on students at Kermanshah University of Medical Sciences (N=4200) from July to August 2020. All students at Kermanshah University of Medical Sciences, including students in the School of Health (n=50), the School of Paramedical Sciences (n=70), the School of Nursing (n=50), the School of Medicine (n=60), the School of Pharmacy (n=40), and the School of Dentistry (n=30) participated in the study. The sample size was estimated to be 300 students via the Morgan table. Participants were selected via stratified random sampling; students were selected from all educational disciplines.

### Data Collection and Descriptive Analysis

Data were collected using the Persian version of the Procrastination Assessment Scale for Students (PASS) by Solomon and Rothblum [21] and the Metacognition Questionnaire-30 (MCQ-30) by Wells and Cartwright-Hatton [22]. The PASS was first developed and used by Solomon and Rothblum in 1984. In this 27-item tool, items 2, 4, 6, 11, 15, 16, 21, 23, and 25 are scored reversely. The scale evaluates procrastination in the following three areas: preparing for an exam (items 1-6), completing assignments (items 9-17), and writing papers at the end of a semester (items 20-25). This scale has been used and validated in previous studies, and its reliability has been confirmed with a Cronbach α of .73 [21]. In this study, its internal consistency was estimated to be 0.84, and its reliability was confirmed with a Cronbach α of .64 [22]. This questionnaire was reviewed and approved in a study by Mortazavi et al [23] in Iran, who conducted a confirmatory factor analysis on an ethnically diverse sample of 345 participants. The MCQ-30 is a 30-item self-report tool consisting of 5 subscales, in which items 2, 4, 6, 11, 15, 16, 21, 23, and 25 are scored reversely. The five factors are cognitive confidence, positive beliefs about worry, cognitive self-consciousness, negative beliefs about the uncontrollability of thoughts and danger, and beliefs about the need to control thoughts. The MCQ-30 showed good internal consistency and convergent validity and acceptable to good test-retest reliability [22].

The data analysis was performed in SPSS (version 20; IBM Corporation) using the Pearson correlation coefficient and regression analysis. The Pearson correlation coefficient was used to estimate the relationships between the study variables. The regression coefficient was used to calculate the predictability of academic procrastination based on metacognitive beliefs.

## Results

The mean age of the students was 21.86 (SD 2.70) years. In terms of sex, 150 (50%) of the 300 students were female. The mean total score of procrastination was 63.67 (SD 4.88), and the mean scores of procrastination in the dimensions of preparation for an exam, completing assignments, and completing homework during the semester were found to be 18.53 (SD 3.05), 25.79 (SD 4.08), and 18.62 (SD 1.99), respectively. Table 1 shows the mean scores of the metacognition variable and its dimensions.

Tables 2 and 3 show the correlation coefficients of academic procrastination, metacognitive beliefs, and their subscales. The results indicated a significant negative correlation between the subscale of positive beliefs of concern and academic procrastination (*r*=–0.16; *P*<.001). In other words, a higher score for academic procrastination was associated with a lower score for the subscale positive beliefs of concern (Tables 2 and 3).

In this study, a simultaneous multiple regression analysis was applied to determine the share of each component of metacognitive beliefs in determining the variance in academic procrastination. As shown in model 1, approximately 10% of the variance in academic procrastination could be predicted based on metacognitive beliefs (model 1: *R*=0.309; *R*^2^=0.095; justified *R*=0.089; *F*_298_=15.63; *P*=.001). The *F* ratio also indicated that academic procrastination could be predicted based on the variable of metacognitive beliefs—something that was statistically significant (*P*=.001; Table 4).

The *F* ratio demonstrated that the regression of the criterion variable (ie, academic procrastination) was significant based on the predictive variables (*P*=.001); in other words, the components of metacognitive beliefs were considered significant. Among the dimensions of the metacognitive beliefs variable, the elements of positive and negative metacognitive beliefs affected the students’ academic procrastination, predicting 10% of their academic procrastination (model 2: *R*=0.324; *R*^2^=0.105; justified *R*=0.090; *F*_298_=6.89; *P*=.001). On the other hand, the component of positive metacognitive beliefs with the β value of –.45 negatively and significantly predicted the students’ academic procrastination (*P*<.001), while the component of negative metacognitive beliefs with the β value of .39 positively and significantly predicted the students’ academic procrastination (*P*<.001).

According to the obtained β coefficients, the component of positive metacognitive beliefs had the most significant contribution in explaining the variance in the students’ academic procrastination (*P*<.001). In terms of predictive power, the components of positive and negative metacognitive beliefs had the highest and lowest ability to predict procrastination, respectively (Table 5).

**Table 1:** Means and SDs of the total scores for academic procrastination.

Components of procrastination	Score, mean (SD)	Minimum score	Maximum score
Total negligence score	63.67 (4.88)	55	75
Preparation for an exam	18.53 (3.05)	13	23
Completing homework	25.79 (4.08)	18	33
Writing end-of-term papers	18.62 (1.99)	15	25

**Table 2:** Means and SDs of the total scores for metacognitive beliefs.

Dimensions of metacognitive beliefs	Score, mean (SD)	Minimum score	Maximum score
Positive concerns	18.47 (4.43)	10	29
Negative metacognitive beliefs	19.44 (4.57)	9	28
Low cognitive efficiency	18.80 (4.00)	12	27
Negative metacognitive beliefs about thoughts	18.47 (3.60)	12	26
Cognitive self-awareness	22.68 (4.45)	15	33
Total score	94.47 (4.45)	58	136

**Table 3:** Correlation analysis (Pearson r and 2-tailed *P* value) among procrastination variables and dimensions of metacognitive beliefs. Correlation is significant at the .01 level.

Variables		Negligence	The total score of metacognitive beliefs	Positive concerns	Negative metacognitive beliefs	Low cognitive efficiency	Negative metacognitive beliefs about thoughts	Cognitive self-awareness
**Negligence**								
	*r*	1	–0.40	–0.16	0.10	–0.03	–0.03	–0.05
	*P* value	—^a^	.001	.006	.10	.51	.66	.42
**The total score of metacognitive beliefs**								
	*r*	–0.40	1	0.85	0.84	0.74	0.78	0.79
	*P* value	.001	—	.001	.001	.001	.001	.001
**Positive concerns**								
	*r*	–0.16	0.85	1	0.63	0.66	0.45	0.59
	*P* value	.006	.001	—	.001	.001	.001	.001
**Negative metacognitive beliefs**								
	*r*	0.10	0.84	0.63	1	0.45	0.65	0.62
	*P* value	.10	.001	.001	—	.001	.001	.001
**Low cognitive efficiency**								
	*r*	–0.03	0.74	0.66	0.45	1	0.45	0.44
	*P* value	.51	.001	.001	.001	—	.001	.001
**Negative metacognitive beliefs about thoughts**								
	*r*	–0.03	0.78	0.45	0.65	0.45	1	0.49
	*P* value	.66	.001	.001	.001	.001	—	.001
**Cognitive self-awareness**								
	*r*	–0.05	0.79	0.59	0.62	0.44	0.49	1
	*P* value	.42	.001	.001	.001	.001	.001	—

^a^Not applicable.

**Table 4:** β coefficients and *t* test values for academic procrastination.

Criterion variable and predictive variable		B^a^ (SD)	β^b^	*t* test (*df*)	*P* value
**Academic procrastination**					
	Constant	55.70 (2.27)	N/A^c^	24.49 (298)	.001
	Metacognitive beliefs	–0.024 (0.017)	.079	–1.42 (298)	.16

^a^Unstandardized β coefficient.

^b^Standardized beta coefficient.

^c^N/A: not applicable.

**Table 5:** β coefficients and *t* test values for metacognitive beliefs.

Criterion variable and predictive variable		B^a^ (SD)	β^b^	*t* test (*df*)	*P* value
**Metacognitive beliefs**					
	Constant	64.52 (1.72)	N/A^c^	37.63 (298)	.001
	Positive concerns	0.454 (0.098)	.412	–4.63 (298)	.001
	Negative metacognitive beliefs	0.416 (0.092)	.389	4.54 (298)	.001
	Low cognitive efficiency	0.169 (0.091)	.138	1.86 (298)	.06
	Negative metacognitive beliefs about thoughts	–0.118 (0.104)	–.087	–1.14 (298)	.25
	Cognitive self-awareness	–0.068 (0.0082)	–.062	–0.83 (298)	.41

^a^Unstandardized β coefficient.

^b^Standardized beta coefficient.

^c^N/A: not applicable.

## Discussion

### Principal Findings

This study aimed to determine the predictability of academic procrastination based on metacognitive beliefs among students in Iran. A significant negative correlation was observed between the subscale of positive beliefs of concern and academic procrastination (*r*=–0.16; *P*<.001). The metacognitive beliefs of the participants predicted academic procrastination. The component of positive metacognitive beliefs with the β value of –.45 negatively and significantly predicted the students’ academic procrastination (*P*<.001), whereas the component of negative metacognitive beliefs with the β value of .39 positively and significantly predicted the students’ academic procrastination (*P*<.001).

Our results showed that a significant negative correlation was observed between the subscale of positive beliefs of concern and academic procrastination (*r*=–0.16; *P*<.001). The results of this study are consistent with the findings of Hayat et al [24], who reported that 28.85% of students have a high level of academic procrastination and that academic procrastination among postgraduate students is very common and has a negative impact on their mental health. Academic self-efficacy positively correlated with academic self-control and negatively correlated with academic procrastination, and academic self-control negatively correlated with academic procrastination. Academic self-control had a completely mediating effect on the influence of academic self-efficacy on academic procrastination. Sex variables moderated the influence of academic self-efficacy on academic self-control and thus significantly moderated the mediating effect of academic self-control. Specifically, academic self-control had a stronger mediating effect on the influence of academic self-efficacy on academic procrastination for female postgraduate students [25].

In addition, the metacognitive beliefs of the participants predicted 10% of academic procrastination. The component of positive metacognitive beliefs with the β value of –.45 negatively and significantly predicted the students’ academic procrastination (*P*<.001), whereas the component of negative metacognitive beliefs with the β value of .42 positively and significantly predicted the students’ academic procrastination (*P*<.001). Among the dimensions of the metacognitive beliefs variable, the components of positive and negative metacognitive beliefs affected the prediction of the students’ academic procrastination. Since obtaining a higher score on this scale was interpreted as having more negative metacognitive beliefs, a positive correlation was denoted between the 2 variables, indicating that the students with more negative metacognitive beliefs procrastinated more often. Based on the correlation coefficients, a reverse association was also shown, suggesting that those who procrastinated more often had more negative beliefs. In this regard, our findings are in line with the results obtained by studies showing a positive correlation between academic procrastination and metacognitive beliefs [26,27].

According to Özer, PhD (unpublished data, 2010), learning new study skills could reduce procrastination, which might be due to the fact that procrastination is a defect in metacognitive strategies. Throughout the literature, procrastination has been perceived as a failure in self-regulation (ie, metacognitive beliefs) by various researchers [28-30].

Considering the theoretical research background and the results of this study, it could be inferred that the high prevalence of procrastination among students necessitates the attention of education officials and planners toward reducing or correcting academic procrastination. Overall, procrastination is a maladaptive behavior and an inefficient with negative consequences. These findings have implications for the better understanding of academic procrastination and the use of academic interventions to correct this issue.

Based on the findings of this study, the following recommendations could be employed to improve learning and reduce academic procrastination. Given the multidimensional nature of metacognitive variables, the dimensions of procrastination should be evaluated along with these variables. Extensive educational programs could be implemented regarding learning positive and beneficial metacognitive beliefs and avoiding negative and harmful metacognitive beliefs. Special attention should be paid to preventive strategies for procrastination by education authorities, planners, and policy makers. Similar studies should be conducted on students in other educational levels, but such studies should use experimental study designs to increase the generalizability of the results and determine causal relationships. The concept of academic procrastination should also be assessed at lower levels of education to take proper measures for reducing procrastination and preventing its negative outcomes in the education and future careers of younger students.

### Limitations

This study compiles the results of a questionnaire and has its own limitations; there is a possibility of bias, exaggeration in estimating features, or memory errors when responding to a questionnaire. Therefore, measuring each of the variables while performing tasks that are closely related to the real-world situation can provide a more realistic view of the relationships being studied. However, we attempted to gather reliable information. Due to the analysis of correlations between variables, there are limitations in explaining variable relationships causally; therefore, conducting research with an experimental design that examines the interventional effect of metacognitive beliefs about procrastination on reducing academic procrastination to confirm and complete the results of this study would be helpful.

### Strengths

We tried to gather and analyze reliable data. A strength of this study was the completion of questionnaires with the presence of the researcher. Furthermore, this topic has not been studied before in Iran.

### Conclusions

A significant negative correlation was observed between the subscale of positive beliefs of concern with academic procrastination (*r*=–0.16; *P*<.001). The metacognitive beliefs of the participants predicted academic procrastination. The component of positive metacognitive beliefs negatively and significantly predicted the students’ academic procrastination (*P*<.001), whereas the component of negative metacognitive beliefs positively and significantly predicted the students’ academic procrastination (*P*<.001). Due to the predictability of procrastination based on metacognitive beliefs, the modification of metacognitive beliefs to reduce procrastination is suggested.

## Abbreviations

MCQ-30: Metacognition Questionnaire-30
PASS: Procrastination Assessment Scale for Students
